# A Multimodal Therapist-Supervised Robotic Gait-Training Platform with Phase-Synchronized Infrared Thermal Biofeedback: A Concept-Level Technical and Translational Healthcare Framework

**DOI:** 10.3390/healthcare14183101

**Published:** 2026-09-20

**Authors:** Rocco Salvatore Calabrò, Aurelio Crespantini, Andrea Calderone, Stefano Troncone

**Affiliations:** 1 IRCCS Centro Neurolesi Bonino Pulejo, 98124 Messina, Italy; roccos.calabro@irccsme.it; 2 ICS Hermitage Maugeri Napoli, 80145 Naples, Italy; aurelio.crespantini@gmail.com; 3 Pandhora S.r.l., 84085 Salerno, Italy; stefano.troncone@pandhora.it

**Keywords:** robotic rehabilitation, exoskeleton, gait training, neurorehabilitation, infrared thermal biofeedback, infrared-A, virtual reality, artificial intelligence, embodiment, concept-level proof-of-concept

## Abstract

**Highlights:**

**What are the main findings?**

- Li-Walk^®^ is presented as a concept-level, therapist-supervised architecture integrating robotic gait guidance, dynamic body-weight support, phase-synchronized IR-A thermal biofeedback, directional audio, semi-immersive visual feedback, body representation, and bounded AI-supported decision support.
- The architecture combines shared system-state coordination with safety-by-design principles and staged validation requirements. Bench performance, patient safety, and clinical efficacy remain unverified.

**What are the implications of the main findings?**

- Multimodal rehabilitation platforms should be verified at both component and system levels, including synchronization, stop hierarchy, clinician override, and the timing of mechanical, thermal, acoustic, and visual events.
- Independent bench, IR-A, software/AI, and human-factors validation should precede authorized human testing and any patient-facing therapeutic claim.

**Abstract:**

Robotic gait technologies can deliver intensive, repetitive, task-oriented stepping, but multimodal platforms require careful integration before clinical testing. This concept-level technical proof-of-concept describes Li-Walk^®^, an investigational fixed robotic gait-training platform combining a treadmill-synchronized lower-limb exoskeleton, dynamic body-weight support, phase-synchronized infrared-A (IR-A) thermal biofeedback, directional acoustic feedback, a semi-immersive display, camera-derived body representation, and artificial intelligence (AI)-supported therapist guidance. The architecture is defined through subsystem interfaces, implementation status, operational states, explicit risk controls, and a staged validation roadmap. No human participants, patient-level data, bench datasets, or statistical analyses were involved; subsystem factory checks did not characterize integrated performance. The installed exoskeleton has four actuated sagittal axes at the hips and knees, with in-series load cells supplying interaction-force inputs for contingent ipsilateral IR-A cueing under reduced guidance. The integrated AI module combines rule constraints with a case-based design, but has no trained statistical model or clinical cohort database; the therapist retains parameter-setting authority. Live video and avatar modes are implemented, whereas independent thermal monitoring and structured recommendation audit trails remain planned. Trigger thresholds, sampling rates, end-to-end latency, exposure limits, and fault responses remain undocumented or unverified. The contribution is a conceptual integration framework that distinguishes implemented functions, declared specifications, and unverified requirements. Independent mechanical, thermal, software, and human-factors validation, followed by appropriately authorized human studies, is required to establish safety, usability, technical reproducibility, and any incremental rehabilitation benefit. The optional thermal branch requires particular safeguards for impaired thermal sensation and cannot be assumed to add clinical value beyond robotic gait training alone.

## 1. Introduction

Gait impairment remains a major determinant of disability after stroke, spinal cord injury, traumatic brain injury, and progressive neurological disease. Its clinical impact extends beyond reduced walking speed, because impaired mobility influences balance, participation, self-efficacy, and the opportunity to practice meaningful activities in daily life. Contemporary neurorehabilitation therefore emphasizes intensive, task-specific, and goal-directed training, delivered with feedback that helps users interpret movement, calibrate effort, and adapt behavior over time [1,2].

Robotic and digital technologies can support these principles by increasing repetition, standardizing assistance, and capturing performance information. Their value, however, depends on clinical interpretability and safe integration within therapist-led care. Technology should preserve motor learning principles and professional judgment. It should help therapists deliver controlled practice while preserving active contribution, transparent feedback, appropriate challenge, and meaningful task context [3,4]. Stroke-related loss of voluntary control makes the distinction between imposed stepping and attempted active movement particularly important: reduced robotic guidance should challenge residual contribution without treating assisted motion as evidence of recovered volition.

A technical proof-of-concept article can contribute before clinical data are available when it clarifies how separate modules should operate together. This is particularly important for robotic gait systems because potential effectiveness is not determined only by the number of steps delivered. It also depends on how the device constrains joint motion, how much voluntary effort is preserved, how sensory information is timed, how failure states are handled, and how the therapist can interpret or interrupt the session. Premature clinical language may obscure these requirements and make later trials harder to design.

Electromechanical gait training and lower-limb exoskeletons have shown promising but heterogeneous effects in neurological rehabilitation [5,6,7,8,9,10]. This variability reflects differences in disease stage, training dose, comparator, device architecture, and control strategy. Fixed robotic gait platforms differ from wearable exoskeletons because the user interacts simultaneously with joint guidance, treadmill motion, body-weight support, and therapist-selected parameters. For this reason, proof-of-concept reporting should describe mechanical constraints, physical human–robot interaction, assistance timing, and safety assumptions before clinical benefit is discussed [11,12,13,14,15,16,17,18].

Virtual reality and multisensory feedback provide complementary mechanisms for making repetitive stepping more understandable and engaging. Semi-immersive displays can supply visual flow and task context while preserving therapist access and environmental awareness. Auditory cueing may reinforce rhythm and step timing, whereas biofeedback can make otherwise hidden performance variables perceptible [19,20,21,22,23,24,25,26,27,28,29,30]. These channels must be synchronized with the mechanical task. A visual scene, sound cue, or thermal event that is delayed, wrong-sided, or dissociated from movement could create perceptual conflict rather than useful rehabilitation feedback.

Avatar-mediated body representation adds a further design layer. A displayed body, live image, or avatar may support visuomotor congruence, mirroring, agency, and body-related engagement, particularly when neurological gait impairment disrupts confidence, body image, or the perceived link between intention and movement [31,32,33,34,35,36,37,38,39,40]. These mechanisms remain hypothesis-generating. Avatar realism, perspective, latency, and movement fidelity should be selected carefully because unrealistic performance or delayed feedback may reduce embodiment, increase frustration, or contribute to cybersickness.

Artificial intelligence (AI)-supported rehabilitation is increasingly discussed as a means of converting movement-related information into therapist-facing recommendations. At this early device-development stage, however, algorithmic support should remain bounded, interpretable, logged, and reversible. The physiotherapist must retain responsibility for goals, progression, parameter acceptance, and interruption, while human-factor engineering should address fitting, alarms, recommendations, and error recovery as safety-critical tasks.

Infrared and thermal stimulation require similarly cautious framing. Biological interpretation depends on source class, wavelength, irradiance, exposure geometry, dose, duty cycle, skin temperature, and safety controls. The Li-Walk^®^ concept uses short-wave infrared-A (IR-A) as phase-linked thermal biofeedback rather than as a verified photobiomodulation intervention. Thermal output is intended to alternate between limbs during the hip-extension propulsive phase and, when robotic guidance is reduced, to be triggered by ipsilateral hip interaction force. This force-based authorization does not establish voluntary neural drive. This strategy may provide a perceivable sensory marker of propulsion, but it requires source characterization, timing validation, and controlled testing before any therapeutic claim is made.

The specific design question is whether force-contingent, ipsilateral thermal cues can be coordinated with robotic stepping, visual body representation, and therapist-facing recommendations within one optional, supervised feedback architecture. Existing robotic gait, virtual-reality, and biofeedback approaches already combine assistance and sensory information [11,12,13,14,15,16,17,18,19,20,21,22,23,24,25,26,27,28,29,30,31,32,33,34,35,36,37,38,39,40]. Li-Walk^®^ adds a proposed thermal-contingency and coordination framework; its incremental value and reliability remain untested. The contribution therefore concerns explicit implementation boundaries and validation requirements, rather than priority of invention or demonstrated therapeutic superiority.

Li-Walk^®^ was conceived within this converging landscape as an investigational fixed robotic gait-training platform integrating a treadmill-synchronized exoskeleton, dynamic body-weight support, phase-synchronized IR-A thermal biofeedback, directional audio, semi-immersive visual scenarios, camera-derived body representation, and AI-supported therapist guidance.

## 2. Materials and Methods

### 2.1. Design and Scope

This project report presents a concept-level technical proof-of-concept and translational design rationale. The term proof-of-concept is used at the level of system architecture, functional integration, design-input consolidation, and future validation planning. It does not denote demonstrated clinical efficacy, patient safety, user acceptability, independent bench verification, or confirmed engineering performance.

No human participants or animals were involved, no clinical intervention was delivered, no patient-level dataset was generated, no adverse event information was collected, and no statistical analysis was performed. The article describes the proposed platform architecture, safety-by-design logic, and validation roadmap needed before human testing or clinical claims can be considered. No formal or informal bench dataset is available. Subsystem mechanical and electrical factory checks were performed as production-conformity checks without recording timing, irradiance, sampling frequencies, or error rates.

The design outputs comprise component mapping, information-flow modeling, risk-domain identification, specification synthesis, and validation planning. They define what subsequent engineering, human-factors, and clinical investigations must measure; they do not constitute an experimental evaluation of the integrated platform.

Generative AI was used in preparing the manuscript and its illustrative material, not to produce technical measurements or validation evidence. ChatGPT (version 5.6; OpenAI, San Francisco, CA, USA) assisted with language editing, technical-text organization, reference checking, and document formatting. Its image-generation function was used to prepare conceptual illustrations, with author-directed figure layout and review of the labels and pathways against the technical description. Depicted individuals and test scenes are illustrative and do not represent study participants, recorded clinical sessions, or completed evaluations. The authors reviewed and edited the AI-assisted material and retain responsibility for its accuracy. This editorial use is distinct from the rule-constrained, case-based decision-support software described within the platform.

### 2.2. Technical Description and Specification Framework

The technical description was organized around the current prototype configuration, its functional modules, its operating envelope, and the interactions among mechanical, sensory, visual, embodied, and digital layers. The objective was to convert a multimodal device concept into a transparent scientific framework that can be audited, verified, and expanded through future studies using controlled technical records.

Numerical values are identified as nominal component or system specifications, clinician-selectable settings, or unverified design requirements. Implemented functions have not undergone independent performance testing. The same principle applies to the infrared (IR) and AI modules. Their intended functions are described without assuming optical dose–response effects, algorithmic validity, clinical safety, or treatment efficacy.

The specification framework was organized around reproducibility and verification readiness. The framework distinguishes implemented functions, nominal specifications, unverified requirements, planned functions, and parameters that are undocumented or not yet characterized. This separation is necessary because a value in a declared specification is not equivalent to a validated performance claim. For example, a speed range must be linked to acceleration, stopping distance, synchronization with joint movement, and eligible user configurations. Likewise, an IR intensity setting must be linked to source output, exposure distance, temperature monitoring, and skin-safety limits. The framework therefore treats the platform as an evolving medical-device concept rather than as a finished clinical system. “To be determined” identifies unavailable parameter values without assigning estimates; “not measured” identifies absent performance data.

The extended engineering requirement matrix, staged validation checklist, and risk-management checklist are provided in Tables S1–S3, respectively.

### 2.3. Overall System Architecture

Li-Walk^®^ (Pandhora S.r.l., Mercato San Severino, Italy) is organized as a fixed gait-training station rather than a freely wearable exoskeleton. The mechanical subsystem comprises a lower-limb robotic exoskeleton, a synchronized treadmill, and a dynamic body-weight support structure. The sensory-output subsystem comprises infrared thermal panels, directional acoustic speakers, and a curved semi-immersive display. A camera-derived body representation places the user within the visual environment, while a touchscreen and AI-supported assistant provide the therapist-facing interaction layer.

All listed modules are physically installed and integrated in the prototype. The control layer derives gait phase from the exoskeleton-imposed pattern. Sampling rates, clock architecture, communication protocol, control-cycle timing, drift management, and packet-loss recovery have not been documented or characterized. IR-A is optional; sensory feedback and AI can be disabled, leaving therapist-controlled mechanical operation, including treadmill and body-weight support (BWS) without the orthosis.

The proposed state model covers setup, calibration, active stepping, pause, controlled stop, fault, and emergency stop. Implemented operator controls include touchscreen and remote pause/stop commands, emergency devices, and manual reset. Automatic stops for excessive deviation or missing periodic therapist-presence confirmation are implemented. Communication loss should disable sensory channels and trigger controlled mechanical stopping, but this response and automatic emitter deactivation on stopping remain unverified requirements. A shared state model therefore specifies intended dependencies without demonstrating synchronized fault tolerance.

### 2.4. Robotic Gait Training and Body-Weight Support Module

The robotic module guides lower-limb movement while the treadmill provides a controllable walking surface. The body-weight support (BWS) structure is intended to reduce loading on the lower limbs and to maintain the user inside the mechanical workspace. This configuration is compatible with repeated, therapist-supervised stepping for individuals who may not be able to sustain full body weight, although clinical testing has not yet been conducted.

The dynamic exercise and static lifting ratings apply to different operating states; the static rating alone does not establish eligibility for dynamic walking exercise.

The exoskeleton has four independently controlled sagittal axes: bilateral hip and knee flexion-extension, each driven by a dedicated electric linear actuator with an in-series tension/compression load cell and a position sensor. The ankle interface is passive, unactuated, and uninstrumented. Adjustable settings include hip range of motion (ROM) and offset, knee flexion ROM with non-negative offset to prevent hyperextension, treadmill speed, orthosis cadence, side-specific guidance, and unloading. Numerical joint limits and position-sensor resolution are not characterized; ±2° ROM accuracy at 100% guidance is an unverified design requirement.

### 2.5. Infrared Thermal Stimulation and Multisensory Feedback

The IR subsystem is designed as a phase-synchronized thermal biofeedback module. In the current configuration, thermal output is provided by independent short-wave IR halogen emitter units dedicated to each lower limb. The emitter carries a manufacturer-declared CE 0044 marking and Class IIa classification. Manufacturer-reported photobiological testing of the emitter refers to DIN EN 62471:2009-03 [41]. Each unit integrates three 200 W halogen lamps, corresponding to 600 W of summed lamp ratings per panel, not a measured irradiance or absorbed dose, with 230 V supply, three reflectors intended to distribute radiation over the target limb, a protective grid, and an electronic timer. Independent characterization remains necessary for emitted spectrum, irradiance, exposure geometry, skin-temperature profile, trigger latency, and fail-safe behavior.

Nominal emitter specifications include a peak near 1.0 µm, a color temperature of approximately 2400–2500 K, and a power rise time below 1 s. These are component specifications: the broadband spectrum has not been independently characterized, and emitter rise time is not detection-to-command or end-to-end cue latency. Jitter, shutdown, and thermal decay are also unmeasured. The module functions as a thermal-sensory biofeedback channel; laser-based or photobiomodulatory mechanisms are not established. Verification requires spectroradiometry or equivalent source characterization, thermal mapping, exposure timing, safety interlocks, and photobiological-risk assessment according to applicable lamp-safety standards.

The minimum infrared characterization protocol is provided in Table S4.

The contingent pathway uses the sign and amplitude of interaction force relative to a passive reference recorded at 100% guidance. Ipsilateral hip load-cell input authorizes the corresponding IR-A panel during hip extension when guidance is below 100%; electromyography (EMG), motor current, and kinematic deviation are not the IR-A trigger inputs. Cells undergo manufacturer calibration and unloaded offset zeroing at session start. Patient-specific calibration is to be determined. Firmware sampling rate, formal feature definition, thresholds, time windows, and false-trigger suppression are not documented or characterized (to be determined). Excessive deviation triggers an orthosis stop rather than authorizing the IR-A cue.

From a physiological perspective, controlled warmth may contribute to body-related feedback by activating cutaneous thermosensory pathways. Warmth perception involves transient receptor potential (TRP) channels and thermally sensitive afferent pathways, but the contribution of this mechanism to robotic gait rehabilitation remains untested. The cue is intended to make a force-associated gait event perceptible; it does not measure voluntary neural drive or establish motor learning. No independent temperature sensors are installed. Exposure geometry, duration limits, and thermal thresholds are to be determined; automatic cutoffs are planned. Future IR-A protocols should exclude impaired or absent thermal sensation until appropriate eligibility rules and safeguards have been established; the branch can be disabled and visual/acoustic feedback retained. These are proposed prerequisites, not screening procedures already performed. Directional acoustic output is treated similarly as a cueing channel whose value depends on timing, laterality, sound-pressure limits, and preserved communication between therapist and user.

### 2.6. Semi-Immersive Virtual Reality, Avatar Representation, and Embodied Interface

The visual subsystem uses a 49-inch curved monitor to present scenarios synchronized with the training task. Because the system is not described as head-tracked, stereoscopic, or fully occlusive, it is classified as semi-immersive virtual reality. This distinction is important because field of view, visual motion, viewpoint stability, and interaction fidelity influence presence, cybersickness, and user interpretation.

Live user video and avatar modes are implemented; a segmented silhouette is planned. An integrated red–green–blue (RGB) camera supplies local processing on the control computer, without video recording or network transmission. Camera model, resolution, and frame rate are undocumented. The therapist can disable the camera, and the system can operate without it.

The intended function of the avatar is not to restore body schema but to create a temporally responsive visual mediator between assisted movement, intention, body perception, and engagement. The displayed body should preserve congruence with the physical event. Excessive delay, exaggerated movement, or an idealized avatar that exceeds the intended assisted action may weaken agency and generate unrealistic expectations.

The embodied interface should be specified with the same rigor as the mechanical components. Perspective, scaling, visual delay, body-part visibility, and the degree to which the representation follows assisted rather than voluntary motion remain to be characterized. Database exchange is limited to session parameters intended to be anonymized; anonymization effectiveness, re-identification risk, access controls, encryption, and deletion policies have not been assessed. Video retention is not applicable to the non-recording mode. These variables affect how the user interprets the displayed body. They also determine which validation measures are appropriate, including presence, body ownership, agency, self-location, perceived effort, and cybersickness.

### 2.7. AI-Supported Therapist Guidance and Adaptive Training Logic

The integrated AI-supported assistant combines a rule layer, which bounds suggestions by therapist-selected limits, with a case-based design intended to retrieve similar profiles and associated session histories. No statistical model has been trained, and the pilot database is not populated by a clinical cohort. Consequently, the intended comparison of recovery trajectories cannot currently support validated recovery-time optimization. Inputs comprise patient profile, session speed, BWS, guidance, pattern, and duration, together with interaction force, ROM, and cadence. Outputs are therapist-facing suggestions and motivational voice feedback.

Input units are newtons, degrees, steps/min, km/h, kilograms or percentage body-weight unloading, percentage guidance, and minutes. Profile fields, similarity metric, feature definitions, preprocessing, update frequency, temporal windows, and missing-data handling are to be determined. The module has no write access to treatment parameters: the physiotherapist accepts, modifies, or ignores suggestions and retains responsibility for selection, goals, fitting, progression, and stopping.

Structured recommendation audit trails are planned but not implemented. Future verification must separately examine rule compliance, case-retrieval validity, unsuitable or incomplete profiles, database bias, and automation bias. Confidence displays and out-of-distribution detection are candidate controls, not current capabilities. Recommendation failure must be assessed independently from the load-cell-based IR-A trigger; manual control is available without the AI module.

The software and AI verification plan is provided in Table S5.

### 2.8. Safety-by-Design, Regulatory Status, and Future Validation Framework

Li-Walk^®^ remains under technical and regulatory development. The integrated platform’s classification, finalized intended purpose, and conformity-assessment status are to be determined. The infrared emitter’s component-level CE designation does not establish certification, safety, or readiness for routine clinical use of the complete platform.

Safety-by-design should be established before human exposure. Relevant domains include mechanical motion, alignment, interface forces, suspension, harness pressure, treadmill coordination, IR exposure, acoustic output, visual stimulation, cybersickness, software state control, electrical safety, cybersecurity, human–machine interaction, AI oversight, and error recovery. Future verification should be aligned with applicable medical-device risk-management and usability frameworks, including risk analysis, essential-performance definition, software lifecycle control, photobiological safety assessment, electrical safety, electromagnetic compatibility, human-factors engineering, and traceability where applicable.

The validation framework was deliberately staged because the combination of mechanical assistance, sensory output, virtual representation, and digital decision support creates interdependent risks. Bench verification should precede any human exposure. Simulated-use testing should precede clinical deployment. Human-factor testing should involve representative therapists and realistic interruptions, not only idealized demonstrations. Regulatory review should consider the complete system and its intended purpose rather than only the exoskeleton frame. The framework also requires documentation of residual risk, because some hazards may be acceptable only under restricted indications, trained supervision, or conservative starting parameters. Future clinician evaluations and human exposure will require applicable ethics review, consent, and data protection. No ethics approval or participant consent is claimed for this non-human, non-experimental report.

## 3. Technical Architecture and Implementation Status

The architecture, implementation matrix, and risk framework constitute concept-level design outputs. They are not experimental performance results and should not be interpreted as evidence of clinical safety, usability, bench-verified performance, or efficacy.

### 3.1. Device-Level Integration

**Figure 1:**
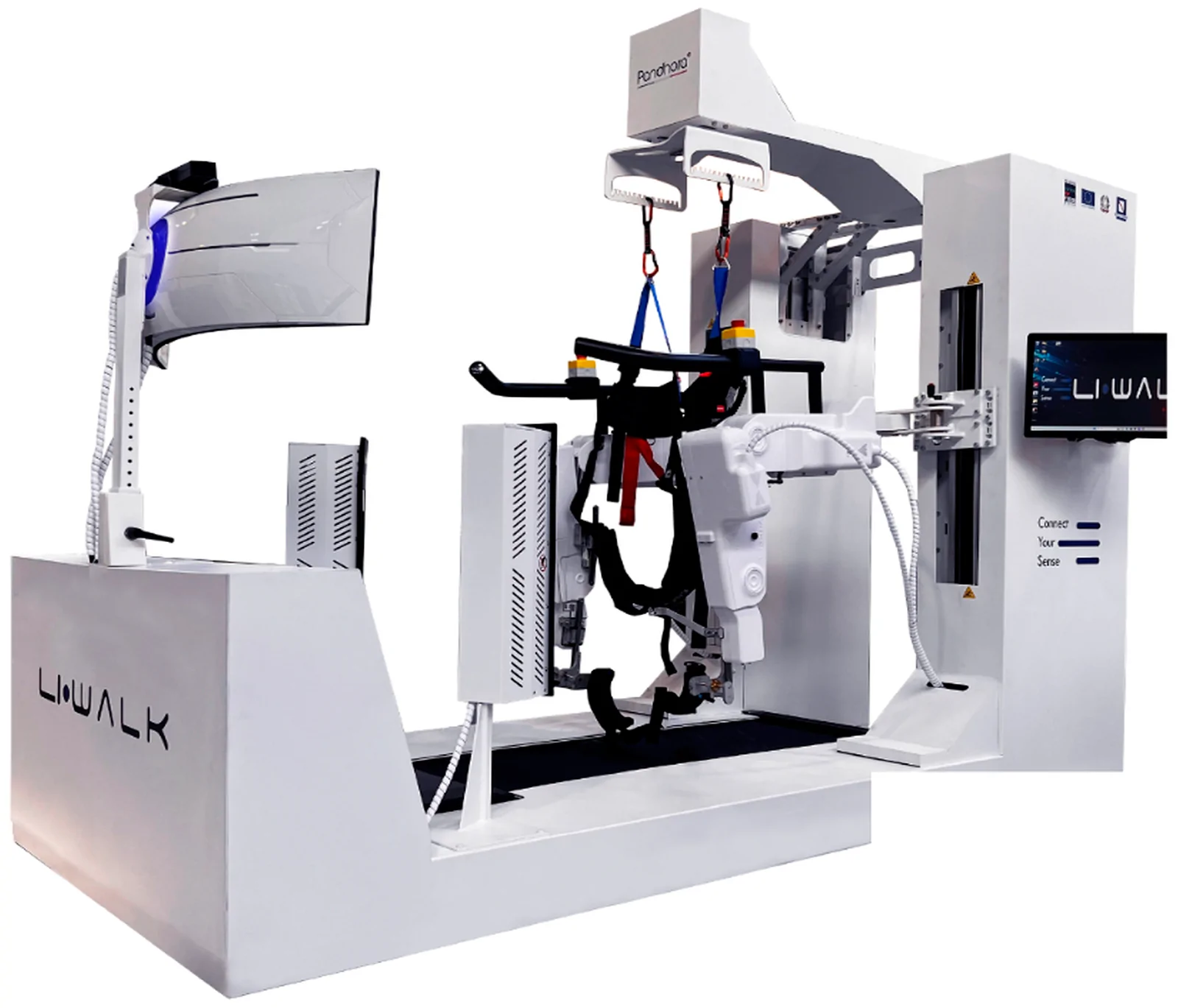
Overview of the Li-Walk^®^ multimodal robotic gait-training platform. The panel shows the fixed robotic station, including the lower-limb exoskeleton, treadmill, dynamic body-weight support structure, curved semi-immersive display, infrared thermal panels, therapist-facing interface, and associated support components. The image is presented as a technical overview of the platform architecture and does not imply clinical validation.

Nine principal functional elements converge on a single supervised gait-training task. The physical configuration of the platform is shown in Figure 1. The robotic exoskeleton, treadmill, and body-weight support form the movement-delivery core. Infrared panels, directional audio, and the curved display form the sensory-output layer. Camera-derived body representation links the physical user to the visual scene. The touchscreen and AI-supported assistant provide the therapist-facing interaction layer.

**Figure 2:**
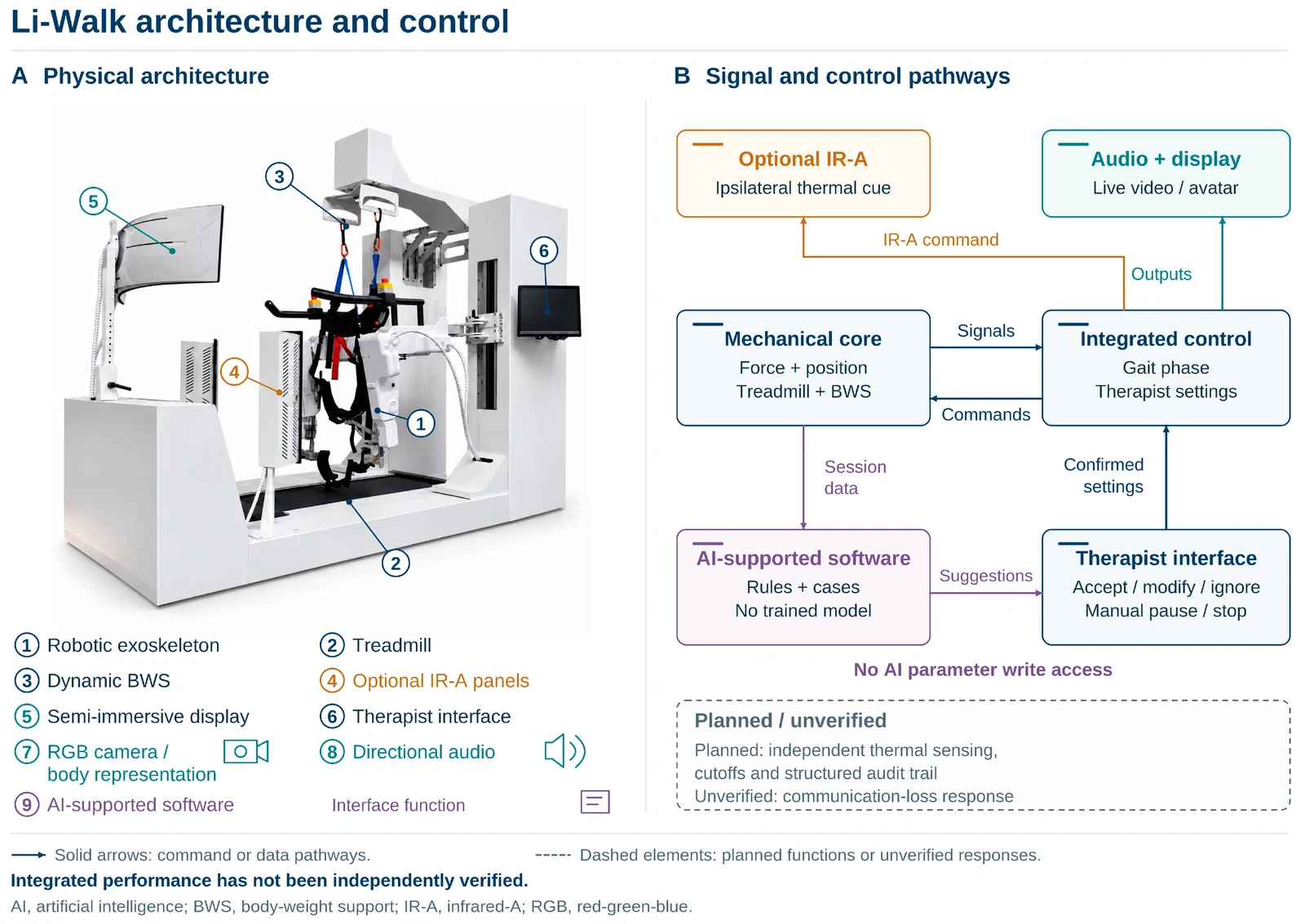
Li-Walk architecture and control. (**A**) Illustrative rendering of the platform and its functional modules. Four active sagittal hip/knee axes use electric linear actuators with in-series load cells and position sensors; ankle interfaces are passive and uninstrumented. Legend entries 7–9 identify the RGB camera/body representation, directional audio, and AI-supported software, respectively; their symbols indicate functions without assigning fixed physical locations. (**B**) Solid arrows distinguish commands, data, and therapist-facing suggestions. Ipsilateral hip force authorizes the IR-A cue during hip extension under reduced guidance. The AI layer combines rules and a case-based design without a trained statistical model or clinical cohort database; only the therapist changes treatment parameters. Dashed elements distinguish planned thermal sensing, cutoffs, and audit trails from the unverified communication-loss response. Independent thermal sensing is absent, and integrated timing, safety responses, and performance have not been independently verified. Blue denotes mechanical, control, and therapist-interface elements; orange denotes IR-A; teal denotes camera, audio, and visual functions; purple denotes AI-supported software and its data/suggestion pathways. Grey dashed elements indicate planned functions or unverified responses.

The corresponding engineering architecture is summarized in Figure 2.

The installed configuration supports mechanical assistance, optional sensory output, and therapist-controlled operation. Integration does not establish common timing accuracy, automatic fault propagation, or thermal stop performance. The state model in Section 2.3 defines these verification requirements; Table 1 separates implementation status from independent performance evidence.

A second design output is the need to classify interactions as mechanical, temporal, perceptual or decisional. Mechanical integration concerns the physical compatibility among harness, exoskeleton, and treadmill. Temporal integration concerns the synchronization of belt speed, joint guidance, sound, visual flow, and thermal activation. Perceptual integration concerns whether the displayed scenario and body representation make the task understandable. Decisional integration concerns how performance information is converted into therapist-facing recommendations. These layers should be validated separately before the complete platform is tested as a rehabilitation intervention.

### 3.2. Therapist-Supervised Functional Model

The therapist-supervised functional loop begins with user positioning, harness adjustment, and mechanical alignment. The therapist selects initial unloading, trajectory, treadmill speed, and guidance force. During stepping, the system acquires device-state and performance-related variables. The mechanical subsystem delivers guided movement, while the sensory subsystems provide phase-linked thermal, auditory, and visual outputs. In the infrared pathway, right–left panels are intended to alternate with the propulsive hip-extension phase, and ipsilateral hip interaction force authorizes the corresponding thermal cue when robotic guidance is reduced. This signal does not demonstrate voluntary participation. The body representation displays a synchronized image or avatar within the selected scenario. The AI software provides a recommendation interface whose case-based clinical utility remains unvalidated. The therapist accepts, modifies, or rejects the recommendation and remains able to pause or stop the task.

**Figure 3:**
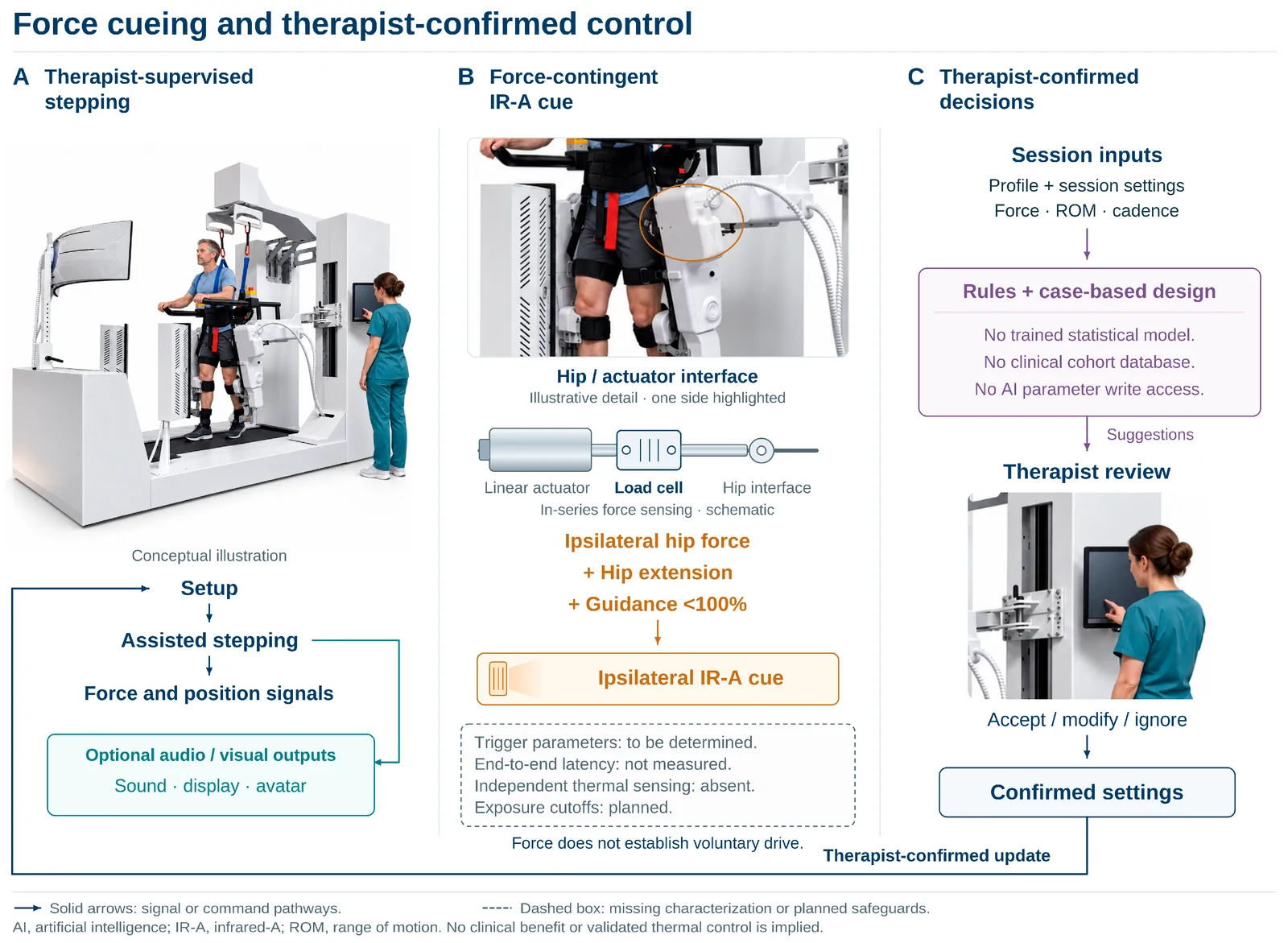
Force cueing and therapist-confirmed control. (**A**) Conceptual illustration of therapist-supervised assisted stepping, signal acquisition, and separate optional audio/visual outputs. The depicted individuals do not represent study participants. (**B**) The illustrative hip detail and schematic sensing chain identify interaction force as the IR-A authorization input. Force sign and amplitude are assessed relative to a passive reference at 100% guidance; ipsilateral hip extension under guidance below 100% permits the corresponding cue. Force is not a direct measure of voluntary neural drive. Electromyography, motor current, and kinematic deviation are not trigger inputs; excessive deviation instead triggers an orthosis stop. (**C**) Session inputs support rule-constrained, case-based suggestions. Only the therapist confirms parameter changes. Trigger parameters remain to be determined, latency is unmeasured, independent thermal sensing is absent, and exposure cutoffs are planned. Automatic emitter deactivation on stopping remains unverified.

The intended information structure is summarized in Figure 3. Closed-loop terminology refers to the force-trigger pathway and therapist-confirmed updates, not verified autonomous adaptation or closed-loop temperature regulation. Independent thermal sensing is absent; trigger accuracy, end-to-end latency, shutdown, and residual heating require bench measurement.

**Figure 4:**
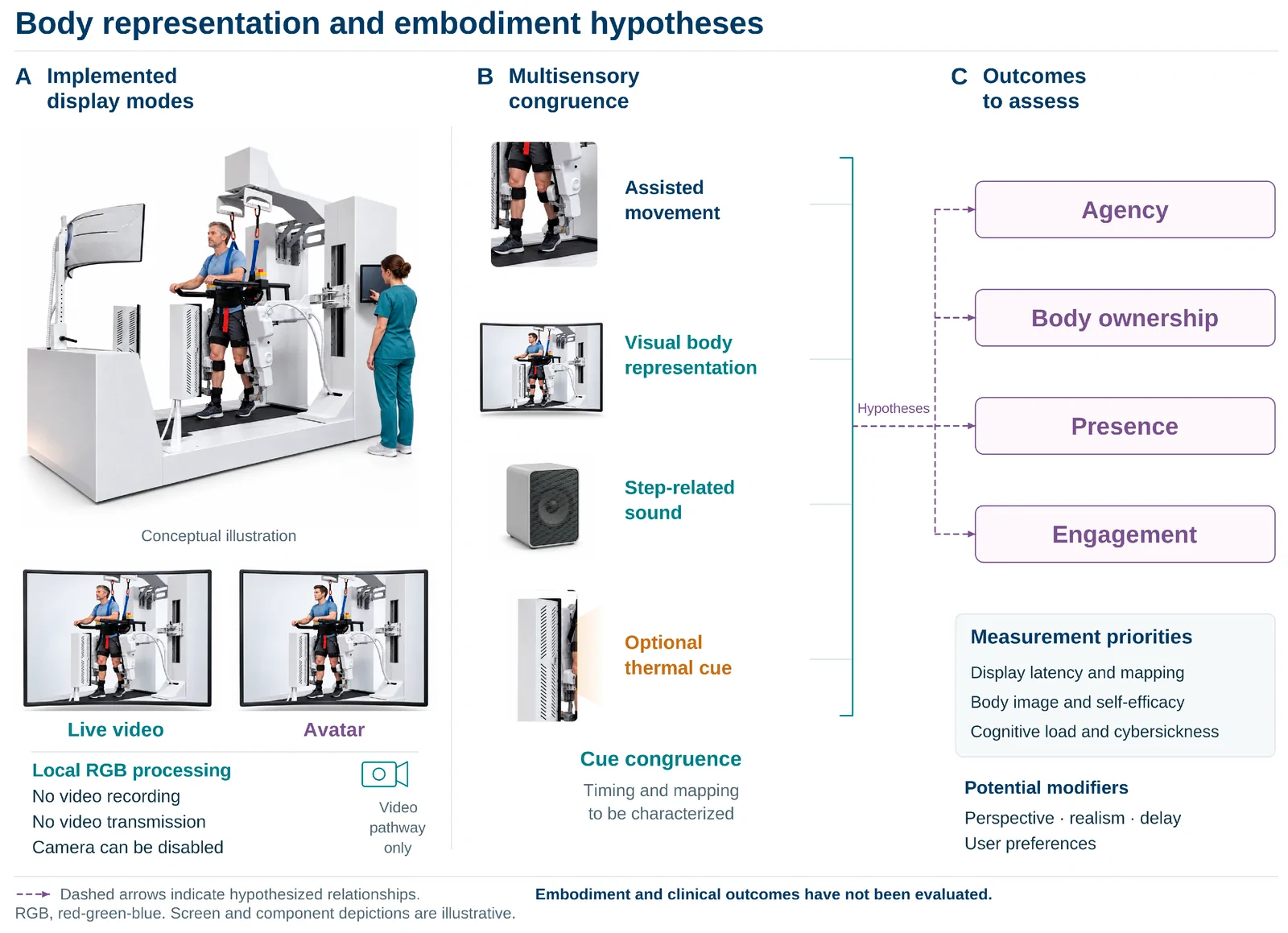
Body representation and embodiment hypotheses. (**A**) Conceptual illustration of the Li-Walk platform and its implemented live-video and avatar modes. The alternative screen examples preserve a comparable supported posture and are illustrative, not study recordings or validated interface renderings. RGB video is processed locally without recording or network transmission, and the camera can be disabled; these properties do not establish protection of all session data. (**B**) Assisted movement, visual body representation, step-related sound, and the optional thermal cue form the proposed multisensory context. Component depictions are illustrative, and timing and movement mapping remain to be characterized. (**C**) Dashed arrows represent hypothesized relationships with agency, body ownership, presence, and engagement, each requiring separate assessment. Measurement priorities and potential modifiers are indicated. Embodiment and clinical outcomes have not been evaluated; no restoration of body schema or clinical benefit is implied.

The related embodiment hypotheses are presented in Figure 4.

The loop also identifies points where clinical supervision must remain active. The therapist must confirm that the user is fitted safely, that the selected unloading and speed are appropriate, that visual and auditory outputs remain tolerable, and that algorithmic suggestions are consistent with the therapeutic plan. The loop is therefore closed through clinician review rather than through autonomous adaptation. Clinical usefulness of the recommendation layer remains to be established.

### 3.3. Technical Specifications and Operational Parameters

The specifications define a preliminary operating envelope for power, speed, user size, load capacity, thermal output, and interface hardware. The nominal parameters and corresponding verification needs are summarized in Table 2. Several clinically important variables are not yet available. These include numerical joint force/torque and speed limits, passive range of motion, alignment tolerances, unloading accuracy, emergency-stop time, treadmill stopping distance, sampling rates, trigger thresholds, thermal-output latency, and synchronization accuracy. Degrees of freedom, actuator type, and interaction-force sensor provenance are specified at the design level in Section 2.4 and Section 2.5.

These missing values limit technical reproducibility and characterization of the operating envelope. Nominal values, tested limits, software-configurable limits, and clinician-selectable limits represent distinct parameter classes. The same distinction is needed for thermal intensity, lamp power, exposure duration, safety cutoff temperature, sound pressure level, visual-scene progression, and AI-supported recommendation boundaries. Current design inputs are sufficient to define a minimum verification plan, but not sufficient to claim verified operation.

The nominal reference session lasts approximately 45 min, comprising 20 min of preparation and 25 min of training; setup/calibration have not been timed separately. The relationship between user mass, dynamic exercise capacity, unloading, and static lifting capacity reflects this same distinction between the static transfer/positioning rating and the load range actively managed by the dynamic lifting system during walking exercise.

### 3.4. Proposed Safety and Risk-Management Framework

The proposed safety framework groups hazards into mechanical interaction, suspension and fitting, treadmill coordination [18,42,43,44,45,46,47,48], infrared exposure, visual and acoustic stimulation [19,20,21,22,23,24,25,26,27,28,29,30,49,50], software control, AI-supported recommendations, electrical performance, cybersecurity, and use error [45,46,47,51,52,53,54,55,56,57]. Table 3 summarizes the corresponding risk domains, potential hazards, candidate controls, and verification evidence. Each control is identified as implemented, planned, or unverified; system-level effectiveness has not been experimentally assessed.

Implemented controls include manual pause/stop, automatic stopping for excessive deviation or absent therapist-presence confirmation, emergency buttons and a magnetic pull cord, a key switch, manual reset, and power-loss release. Emergency operation stops the orthosis and treadmill while retaining support, but response times and single-fault performance have not been measured [18,42,43,44,45,46,47]. Independent temperature monitoring, thermal cutoffs, cue-duration timeouts, and automatic IR-A deactivation require implementation or verification [42,43,49]. Supervision cannot replace these engineering safeguards.

The embodied-feedback pathway introduces a related human-factor domain because visual representation may alter agency, body-related engagement, and the interpretation of assisted movement [31,32,33,34,39,40]. The model remains hypothesis-generating and requires direct measurement of presence, agency, body ownership, self-location, body image, motor self-efficacy, motivation, and cybersickness [19,20,21,22,23,24,30,31,32,33,34,39,40,47].

The risk-control matrix also highlights that mitigation must be distributed across design, software, clinical procedure, and training [18,42,43,44,45,46,47,51,52,53]. A warning label is not an adequate control for excessive joint force if a mechanical limit can be implemented [42,43,44]. Therapist supervision is not an adequate control for thermal exposure if temperature monitoring and cutoffs are feasible [42,43,49]. Conversely, software interlocks cannot replace careful fitting when anatomy and impairment vary across users [18,44,47]. The safer development pathway combines engineering controls with clinician workflow, documentation, and competency-based training [42,43,44,45,46,47]. A stuck-on IR-A command could prolong exposure after mechanical stopping; absent sensation could conceal overheating. An ipsilateral authorization rule does not automatically detect wrong-sided output. An inappropriate case-based suggestion may remain within nominal limits yet be unsuitable for a particular impairment, requiring clinician review and future usability testing.

### 3.5. Future Validation Matrix

The resulting development pathway contains six evidence gates. Specification freeze and traceability establish the intended use, system boundaries, requirements, and risk controls. Bench verification tests loads, alignment, motion limits, electrical performance, and treadmill–exoskeleton coordination. Module verification characterizes IR-A thermal output, right–left alternation, phase timing, software integrity, cybersecurity, and AI-supported functions. Human-factor testing evaluates fitting, interface comprehension, alarms, workload, and foreseeable misuse in simulated use. Authorized human testing then progresses from conservative tolerability assessment to first-in-human safety and feasibility studies. Table 4 organizes these gates, validation domains, suggested methods, and progression criteria. Controlled clinical research becomes appropriate only after these earlier gates have been completed. Expert appraisal is proposed within the human-factors gate: clinicians and rehabilitation engineers should observe documented demonstrations or simulated-use tasks and independently rate feasibility, clinical relevance, burden, and unresolved hazards. No expert evaluation has yet been conducted.

**Figure 5:**
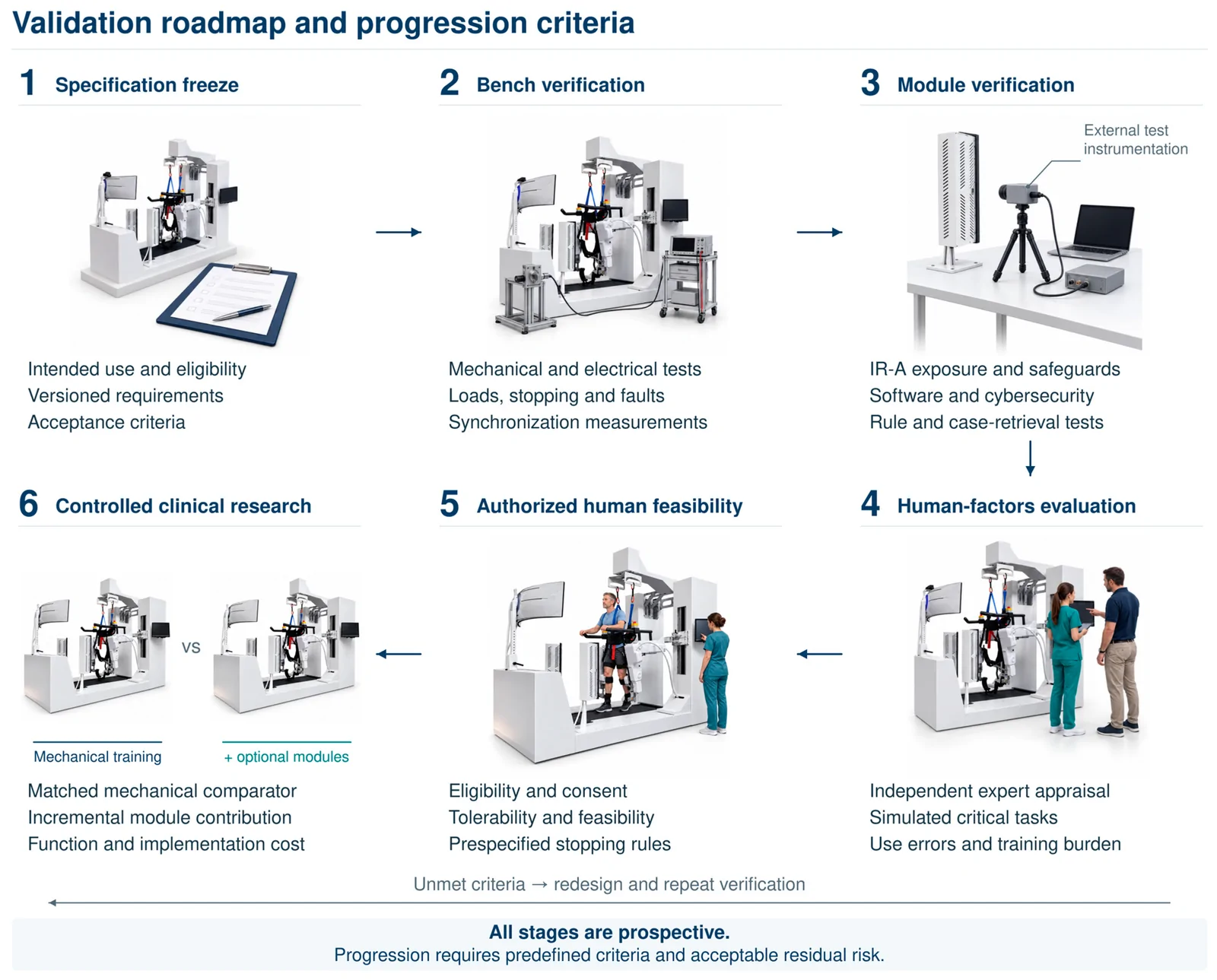
Validation roadmap and progression criteria. Six prospective evidence gates lead from specification freeze through bench and module verification, human-factors evaluation, authorized human feasibility, and controlled clinical research. Arrows indicate conditional progression; unmet criteria require redesign and repeat verification. All scenes are conceptual illustrations of future activities, not documentation of completed testing or enrolled participants. The thermal sensor represents external test instrumentation proposed for module verification, not an installed Li-Walk component. Expert appraisal remains prospective. Human exposure requires adequate technical readiness, acceptable residual risk, informed consent, and applicable ethical and regulatory authorizations. The final comparison depicts the same mechanical platform with optional modules disabled or enabled to assess their incremental contribution, without implying efficacy or superiority. No stage is represented as completed.

Progression should be evidence-gated rather than calendar-driven. Failure at one stage should lead to redesign and repeat verification before exposure is expanded. The same staged logic is condensed graphically in Figure 5. Multimodal combinations can be scientifically valuable only when each component is characterized and the combined intervention is tested through controlled methods.

## 4. Discussion

### 4.1. Healthcare Relevance of a Multimodal Robotic Gait Platform

The architecture raises testable questions about feedback during assisted stepping. A synchronized scenario can give direction and context to treadmill motion. Step-related audio can make temporal events perceptually salient. Phase-synchronized thermal input may add a controllable body-related cue that is spatially and temporally aligned with the limb engaged in propulsion. When guidance force is reduced, contingent activation of the thermal panel could provide a perceivable marker of an interaction-force event during assisted stepping, without establishing that the event reflects voluntary motor drive. These possibilities remain hypotheses for future testing rather than clinical effects established by the current article. AI-supported rehabilitation, intent recognition, and adaptive decision support remain active but incompletely validated areas, and their translation depends on bounded outputs, clinician acceptance, and implementation feasibility [59,60,61,62,63,64,65].

This integration also raises an important methodological implication. When the platform eventually enters human testing, the complete system should not be evaluated only as a black box. A single-arm exposure study may be appropriate for initial safety and workflow assessment, but later studies should determine whether robotic guidance, infrared input, acoustic cueing, avatar feedback, and AI-supported recommendations each provide distinct value. Human-factor engineering, usability evaluation, and human-in-the-loop optimization are therefore not secondary implementation details; they are part of the safety and effectiveness pathway for rehabilitation technology [66,67,68,69,70]. Infrared effects, warmth perception, photobiological safety, and thermal-stimulation paradigms require equally specific validation rather than generic therapeutic assumptions [71,72,73,74,75,76].

### 4.2. Avatar-Mediated Embodiment, Mirroring, and Psychophysical Integration

Body representation is one of the most distinctive conceptual features of the platform. It should not be treated merely as a screen effect. A visually responsive body can mediate between guided movement, the user’s intention, proprioceptive input, and the perception of acting in an environment. When the displayed body moves at the expected time and from a plausible perspective, the user may experience a stronger link between the assisted step and personal action. This link is relevant to agency, body ownership, and confidence in movement, but it must be measured rather than assumed. Broader work on multimodal neurotechnology and current mapping of AI in rehabilitation support the need to evaluate combined interventions as separable components before attributing effects to the whole platform [77,78].

Mirroring in this context is broader than conventional mirror therapy. The platform can display the user’s own image or an avatar while the exoskeleton constrains and assists gait. The representation can therefore provide a coherent view of an action that may be difficult to perceive from the first-person physical perspective. The psychological and physical domains are closely connected because neurological gait impairment can alter self-efficacy, body image, and trust in the affected limb. Fear of falling or movement may reduce participation even when physical support is available.

Design cautions are essential. Excessive delay can disrupt agency. A highly idealized avatar can increase mismatch between observed and felt movement. Visual motion can provoke discomfort or cybersickness. Camera-based projection may raise privacy concerns and can expose clothing, assistive straps, or other details that users do not wish to display. Future studies should directly measure presence, agency, body ownership, self-location, body image, movement self-efficacy, fear of movement, motivation, and cybersickness.

The avatar pathway should therefore be designed with user involvement and usability engineering from the earliest stages. Embodiment cannot be optimized only by increasing realism. Some users may prefer a faithful image of the body, while others may prefer an abstract representation that reduces self-consciousness. A therapist may need to adjust realism, perspective, and feedback intensity according to cognitive load, fear of movement, and session goals. These principles are directly applicable to avatar perspective, scenario relevance, feedback intensity, and therapist control.

### 4.3. Translational, Regulatory, and Human-in-the-Loop Implications

Translation requires the platform to remain human-in-the-loop at both the control and governance levels. A rehabilitation device can benefit from iterative measurement and individualized parameter search, but the objective function, constraints, and user burden must be explicit. This principle does not justify autonomous treatment selection. It supports a structured process in which the system estimates performance, the therapist interprets the estimate, and any adaptation occurs within verified limits.

Regulatory evidence must reflect the complete system. Mechanical verification alone cannot establish safe use when the device includes thermal output, semi-immersive visual content, audio, data logging, and algorithmic recommendations. Usability evaluation should begin during simulated use because risks can arise from fitting sequences, ambiguous labels, alert fatigue, mode confusion, and incorrect recovery from faults. Data governance should be established before shared databases or adaptive models are activated.

Early-stage device papers can inform development by reporting technical rationale, prototype architecture, limitations, and explicit validation gates [79,80,81,82,83]. Clinical implementation additionally depends on space, installation, maintenance, and staff competence. The nominal dimensions are approximately 3.3 × 1.6 × 2.5 m, with a mass of approximately 1000 kg, requiring a suitable room and floor assessment. Manufacturer-led training and preventive maintenance are specified, but training hours, cleaning time, independent operator competency, and uptime have not been quantified. Acquisition cost is unavailable; the stated €70–150 session charge is not an estimate of cost-effectiveness.

Validation of AI-supported functions should not be deferred until clinical trials. Safe healthcare applications require evaluation before deployment, and reviews of explainability, validation, and ethical requirements in medical AI emphasize accountability, transparency, non-maleficence, and protection from hidden bias [84,85,86,87]. These principles are especially relevant when the system may compare a user with a database or infer performance quality from movement variables. The future case database, population coverage, recovery-outcome definition, similarity rules, and missing-data handling require specification before clinical recommendations can be evaluated.

### 4.4. Novelty and Future Directions

The novelty of Li-Walk^®^ does not depend on presenting its individual components as unprecedented [11,12,13,14,15,16,17,18,19,20,21,22,23,24,25,26,27,28,29,30,31,32,33,34,35,36,37,38,39,40,59,60,61,62,63,64,65,66,67,68,69,70,71,72,73,74,75,76]. Lokomat studies already combine interaction-force biofeedback with virtual tasks, while GRAIL couples an instrumented treadmill with an interactive virtual environment [88,89]. These platforms demonstrate prior multimodal integration, although their mechanics, feedback, and tested populations differ from Li-Walk^®^. The narrower proposition here is optional, ipsilateral, force-contingent IR-A cueing integrated with body representation and therapist-controlled recommendations. Lattice pneumatic wrist actuators and perpendicularly enfolded textile actuators illustrate alternative approaches to compliant assistance [90,91]; their component findings do not validate rigid gait-platform control or IR-A safety. The reported architecture is therefore a testable coordination design, not evidence of superiority.

The thermal pathway differs from continuous heating because its intended signal is a gait-associated event. This informational role requires preserved perception and reproducible timing; passive phase cues cannot establish active contribution. Visual body representation and step-related sound may convey complementary information, but redundant or conflicting channels may increase cognitive burden. Their combined value must be compared with the same mechanical training without these additions. The recommendation interface remains therapist-controlled, and its lack of a clinical case database precludes claims of optimized recovery [59,60,61,62,63,64,65,66,67,68,69,70].

Future development should first establish whether this integrative novelty is technically real and reproducible. Requirements should be frozen for a defined system version, after which bench verification should quantify alignment, joint travel, treadmill–exoskeleton coordination, unloading response, emergency stopping, and fault propagation. Multimodal timing tests should then measure end-to-end latency, jitter, right–left cue accuracy, and the time required for thermal, acoustic, and visual outputs to cease after a pause or fault. The IR-A subsystem requires independent spectral, irradiance, exposure, and skin-temperature characterization, while software and AI verification should address input provenance, missing-data behavior, case-retrieval validity, unsuitable profiles, planned audit trails, and fault behavior [42,43,44,45,46,47,48,49,50,51,52,53,54,55,56,57,58,71,72,73,74,75,76]. Advancement should depend on predefined acceptance criteria rather than completion of a calendar stage.

The next stage should evaluate the platform with clinicians and simulated users before patient exposure. Formative studies should examine fitting, calibration, parameter selection, alarm interpretation, recommendation review, interruption, and recovery from realistic errors. Summative human-factors testing should determine whether intended users can complete safety-critical tasks without unacceptable use-related risk. Co-design should also address the acceptability of thermal sensation, sound mapping, avatar realism, perspective, privacy, and cognitive load. These studies are necessary because a technically functional module may still be unsuitable when combined with other demands in a supervised rehabilitation session. The clinician usability protocol proposed for this stage is provided in Table S6. Structured ratings should address usefulness, user support, interface clarity, and learning burden alongside observed critical-task errors; disagreement between engineering feasibility and clinical relevance should be reported.

Only after completion of the required technical, regulatory, and usability gates should authorized first-in-human testing be considered. The initial protocol should prespecify a primary feasibility endpoint and focus on fitting feasibility, tolerability, session completion, thermal and visual comfort, operator workload, emergency procedures, data capture, and device reliability rather than functional recovery. Subsequent controlled feasibility studies should compare the complete multimodal configuration with a stabilized robotic-gait condition lacking the additional sensory and AI-supported modules. Component-withdrawal, crossover, or factorial designs may then help determine whether phase-linked thermal cueing, avatar feedback, directional audio, or therapist-supervised digital adaptation make a distinct contribution. Mechanistic outcomes may include kinematics, electromyography, active contribution, perceived agency, embodiment, and movement self-efficacy, but clinical efficacy should not be inferred from these intermediate measures. Matched training dose and therapist contact are needed to isolate added-module effects; sample-size calculations require a prespecified endpoint and justified precision or effect assumptions, which are unavailable here. No clinical analysis was performed.

Later pragmatic research should determine whether the integrated platform can be implemented sustainably in rehabilitation services. Relevant outcomes include training and supervision requirements, setup time, therapist workload, throughput, maintenance, cybersecurity, data governance, accessibility, costs, and equitable eligibility. Every future report should identify the tested hardware and software version, IR-A settings, treadmill speed, unloading policy, avatar mode, acoustic mapping, AI configuration, and criteria for parameter changes. This configuration-level transparency will allow the evidence base to accumulate without treating different versions as the same intervention. Accordingly, the future research program should progress from independently verified integration, through human-factors and early safety testing, to comparative and implementation studies, while maintaining a clear boundary between engineering novelty and demonstrated patient benefit [79,80,81,82,83,84,85,86,87,92]. Technical variables (timing, force, ROM), safety variables (exposure and fault response), experiential variables (agency and workload), functional outcomes, and implementation costs should be analyzed as distinct domains. Current force/position sensing does not itself supply validated clinical outcomes; thermal mapping, EMG, and patient-reported measures require additional instruments or authorized studies.

### 4.5. Limitations

The primary limitation is the absence of empirical validation. The prototype has not been evaluated in patients, and no conclusions can be drawn about tolerability, adverse events, usability, biomechanical performance, engagement, or clinical efficacy. The analysis is descriptive and conceptual rather than experimental. Several specifications that are essential for reproducibility remain unavailable, including exposure limits, sensor sampling rates, trigger definitions, timing tolerances, and AI validation evidence. Installation and factory conformity checks cannot establish repeatability, synchronization reliability, or clinical relevance. Unspecified parameter values remain to be determined. Missing measurements and the absence of expert appraisal limit technical reproducibility and assessment of clinical relevance.

A further limitation is the absence of independent verification of the functional descriptions and nominal specifications. The system is associated with commercial and intellectual-property interests disclosed in the Patents and Conflicts of Interest sections, which creates a potential source of interpretive bias. Nominal specifications cannot establish measured performance, and clinical or comparative inferences require independent verification. Developer involvement may influence specification selection, interpretation, and emphasis on favorable use scenarios. Independent metrology, external human-factors evaluation, and prospective reporting of failures are therefore necessary. Local non-recording video reduces one data pathway but does not resolve consent, session-data security, or re-identification concerns. The absence of thermal monitoring and a populated clinical case database further limit the current claims.

## 5. Conclusions

Li-Walk^®^ represents a concept-level technical proof-of-concept for a multimodal fixed robotic gait-training platform. Its architecture combines guided lower-limb movement, synchronized treadmill walking, dynamic BWS, IR-A thermal stimulation, directional acoustic feedback, semi-immersive virtual scenarios, camera-derived body representation, and AI-supported therapist guidance. The central design proposition is that these modules should operate as a coordinated, human-supervised system rather than as independent additions.

The contribution is the separation of reported implementation, nominal specifications, and unverified requirements within a staged validation framework. Independent bench characterization, thermal safeguards, software verification, and clinician human-factors assessment remain necessary before appropriately authorized human investigation. Subsequent controlled studies must determine whether optional thermal, visual, acoustic, and recommendation modules add benefit beyond a comparable robotic-gait condition. No conclusion about clinical efficacy, safety, usability, economic value, or superiority follows from the present design-level description.

## 6. Patents

The Li-Walk^®^ platform is associated with European Patent No. EP3982901, titled “Infrared ray equipment for robotic rehabilitation on a treadmill, having flexible pelvic attachment”, granted on 12 July 2023 from application No. 20743764.1. The listed inventors are Stefano Troncone, Alexander Troncone, and Chrystel Troncone, with Pandhora S.r.l. as patent holder. The platform is also associated with Italian Patent No. IT202019000001812, Italian patent application/deposit No. DEP. 202025000003655, registered designs Nos. 402020000000841-402020000001342 and registered trademark No. 302019000053934.

## Figures and Tables

**Table 1:** Core components of the Li-Walk^®^ multimodal robotic platform. The table separates implementation status, intended functions, and independent verification needs; numerical specifications are presented separately.

Module	Current Implementation	Intended Functional Role	Current Validation Status
Fixed robotic exoskeleton	Installed: bilateral sagittal hip/knee actuation; electric linear actuators, in-series load cells, and position sensors; passive ankle interface.	Guide repeated hip and knee movement within therapist-selected parameters.	Four active axes specified. Joint limits, sensor resolution, alignment, and ROM accuracy remain uncharacterized or unverified.
Synchronized treadmill	Installed: treadmill coordinated with exoskeleton-imposed stepping; independent therapist control.	Provide controlled stepping speed and repeated gait cycles.	Speed envelope reported. Phase synchronization, stopping distance, and fault behavior require bench testing.
Dynamic body-weight support	Installed: dynamic lifting and unloading system with load cell; separate static and dynamic modes.	Reduce lower-limb loading and support upright gait practice.	Dynamic and static capacities reported. Load accuracy, control response, harness pressures, and mode limits require verification.
Phase-synchronized short-wave infrared thermal biofeedback	Installed, optional: independent right/left halogen IR-A panels. Each side is authorized by its ipsilateral hip load-cell input during reduced-guidance hip extension.	Provide a phase-associated thermal cue contingent on ipsilateral interaction force during reduced-guidance hip extension.	Trigger accuracy, timing, spectrum, and exposure unverified. Independent temperature sensing is absent; exposure limits/cutoffs are planned or undefined.
Semi-immersive visual display	Installed: curved semi-immersive monitor with task-related scenarios.	Provide visual flow, context, task cues, and engagement.	Display size documented. Field of view, refresh rate, optical-flow gain, synchronization, and cybersickness profile require testing.
Camera-derived body representation	Installed: live-video and avatar modes; camera can be disabled. Segmented silhouette is planned.	Support mirroring, body-related feedback, agency, and embodied engagement.	Video is processed locally without recording or network transmission. Mapping, latency, privacy protections, and embodiment require evaluation.
Directional acoustic feedback	Installed: right/left speakers provide corresponding step-related sounds.	Provide rhythm, event-related cueing, and environmental coherence.	Timing, sound-pressure limits, laterality, alarm priority, and communication preservation require verification.
Therapist touchscreen interface	Installed: touchscreen and remote control for therapist parameter selection, pause, and stop, independent of AI.	Support clinician control, monitoring, alarm handling, and override.	Hardware size reported. Usability, permissions, alarm hierarchy, logging, and foreseeable misuse require human-factors testing.
AI-supported assistant and performance monitoring	Integrated software: rule-constrained suggestions and case-based design; pilot database without a clinical cohort; no trained statistical model.	Display parameter suggestions and motivational voice feedback. The therapist accepts, modifies, or ignores suggestions; AI has no parameter write access.	Similarity, feature definitions, missing-data handling, and updates are not formalized. Clinical usefulness unvalidated; structured audit trail planned.

Abbreviations: AI, artificial intelligence; ROM, range of motion. Values describe the current implementation and are not independently verified performance data.

**Table 2:** Current prototype technical specifications and operational parameters. Available values are shown together with the verification needed before they can be treated as validated performance specifications.

Domain	Declared Specification or Implementation Status
Electrical supply	230 V AC, 50/60 Hz
Total system power	2 kW standard; up to 3.5 kW with IR modules (nominal electrical power; not independently measured)
Training speed with exoskeleton	0.1–3.2 km/h (manufacturer-declared; not independently measured)
Treadmill speed without exoskeleton	0.1–10 km/h (manufacturer-declared; not independently measured)
Dynamic exercise capacity	85 kg: dynamic lifting/unloading rating during exercise; user-mass/unloading combinations require verification
Static lifting capacity	135 kg: static transfer/positioning rating; does not establish dynamic walking eligibility
Stated user height	<200 cm
Stated user body mass	<135 kg (declared anthropometric specification; subject to dynamic mode/load limits)
Therapist interface	24-inch touchscreen
Semi-immersive display	49-inch curved monitor
Infrared output	Three 200 W lamps per panel (600 W summed ratings); nominal peak near 1.0 µm; 2400–2500 K; manufacturer-declared full power < 1 s. These values are not measured irradiance, exposure dose, or end-to-end latency.
Reference session workflow	Approximately 45 min: 20 min preparation and 25 min training (nominal session structure; not measured on a clinical series)
Infrared activation logic	Implemented force-contingent ipsilateral cue under <100% guidance during hip extension. Thresholds, sampling, time window, and false-trigger suppression: to be determined (firmware values undocumented/uncharacterized).
Infrared safety features	Manufacturer-reported emitter grid, timer, CE 0044 marking and Class IIa classification, and DIN EN 62471:2009-03 testing [41]. No independent temperature sensors installed. Exposure limits and cutoffs: to be determined; automatic stop-state deactivation unverified. Component status does not certify Li-Walk.
Performance inputs described for AI support	Patient profile; speed (km/h), BWS (kg or % body weight), guidance (%), pattern, duration (min), interaction force (N), ROM (degrees), cadence (steps/min). Formal features and similarity metric: to be determined.
AI-supported outputs	Therapist-facing suggestions and motivational voice feedback. Rule layer plus case-based design; no trained statistical model or populated clinical cohort database; no AI write access to session parameters.

Abbreviations: AC, alternating current; AI, artificial intelligence; IR, infrared; ROM, range of motion. Values are nominal specifications and have not been independently verified. The 85 kg dynamic exercise capacity and the 135 kg static lifting capacity correspond, respectively, to the active walking-support range and the patient transfer/positioning rating of the dynamic lifting system.

**Table 3:** Proposed safety-by-design and risk-control framework. The matrix distinguishes implemented controls from planned controls and unverified requirements; integrated risk mitigation has not been experimentally verified.

Risk Domain	Potential Hazard	Controls and Implementation Status	Verification Still Required
Anthropometric fit and joint alignment	Joint-axis mismatch, cuff pressure, pain, skin injury, or forced range of motion.	Implemented: adjustable hip/knee settings and stop for excessive deviation. Planned/undefined: validated segment-fit limits, alignment tolerances, pressure limits, and fitting checklist.	Fit studies across intended anthropometry, pressure mapping, mechanical travel verification, simulated misuse [18,42,43,44,47].
Body-weight support and lifting	Overload, unintended descent, harness injury, inaccurate unloading, or unsafe transition.	Implemented: load-cell-based support and operator-controlled modes; support retention on emergency stop. Unverified: load accuracy, transitions, and fault response. Design status of additional restraints/alarms is not documented.	Static and dynamic load testing, sensor accuracy, fatigue testing, single-fault analysis, transition testing [18,42,43,44,45,48].
Treadmill–exoskeleton synchronization	Belt and joint-phase mismatch, stumbling forces, abrupt acceleration, or instability.	Implemented: coordinated treadmill/orthosis operation. Unverified requirement: sensory-channel shutdown and controlled mechanical stop on communication loss. Shared-clock, drift, and recovery details remain undocumented.	Latency and jitter measurement, tolerance definition, fault injection, stopping-distance testing [18,42,43,44,45,46].
Contingent thermal biofeedback logic	Delayed, false-positive, or false-negative activation may reinforce the wrong motor event or create misleading perception of active participation.	Implemented: ipsilateral hip-cell authorization and manual exclusion. Undocumented/uncharacterized: force feature, threshold, time window, and false-trigger suppression. Unverified: pause/stop lockout; wrong-sided output is not automatically detected.	Bench and simulated-use trigger testing, latency and jitter analysis, false-trigger rate, state-machine verification, and clinician review of feedback meaning [42,43,49].
Unexpected robotic motion	Collision, excessive joint torque, entrapment, or start without readiness.	Implemented: excessive-deviation stop, therapist-presence confirmation, key switch, emergency devices, and manual reset. Unverified: response times, numerical force/torque limits, and single-fault performance.	Essential-performance testing, startup and recovery-state testing, emergency-stop accessibility, and response [18,42,43,44,45].
Infrared thermal exposure	Stuck-on cue, residual heating after stop, hotspots, wrong-sided output, repeated exposure, or unrecognized injury with impaired thermal sensation.	Implemented: branch disable and emitter grid/timer. Absent/planned: independent temperature sensing, screening, and exposure cutoffs. Undefined: dose/distance limits, thresholds, and maximum cue duration. Unverified: automatic IR-A stop-state deactivation.	Spectral and irradiance characterization, thermal mapping, prolonged-use and fault tests, right–left output verification, cutoff testing, and worst-case exposure analysis [42,43,49].
Visual environment and body representation	Cybersickness, disorientation, visual strain, fear, or embodiment mismatch.	Implemented: live-video/avatar modes and camera disable. Planned: validated perspective/latency limits, graded exposure, and symptom assessment; video is locally processed without recording or network transmission.	Latency testing, display characterization, healthy-volunteer tolerability where authorized, validated symptom measures [19,20,21,22,23,24,31,32,33,34,39,40,47].
Directional audio	Excessive sound level, distraction, masking of alarms, or misleading timing.	Implemented: directional step-related sound and therapist control. Planned/unverified: sound-pressure limits, alarm priority, timing limits, and communication-preserving alternatives.	Sound-pressure measurement, alarm audibility, timing tests, usability evaluation [27,28,29,50].
Software, data, and cybersecurity	Incorrect parameter display, data loss, unauthorized access, corrupted update, or unsafe state.	Implemented: software interface and manual operation without AI. Planned: structured audit trail. Undefined/unverified: session-data encryption, retention/deletion, secure update, recovery, and access-control validation.	Unit, integration, and system tests; cybersecurity assessment; traceability; rollback and recovery testing [42,43,51,52,53].
AI-supported recommendations	Inappropriate suggestions from dissimilar or incomplete profiles, biased case selection, unavailable recovery histories, or automation bias despite nominally bounded outputs.	Implemented: rule constraints, therapist decision, and no AI parameter write access. Planned: structured audit trail and validation of case retrieval. Confidence/rationale displays and out-of-distribution detection are candidate controls, not established capabilities.	Rule-boundary and no-write-access tests; similarity/feature definition; prospective database quality and subgroup assessment; unsuitable-profile scenarios; override, use-error, and logging evaluation [54,55,56,57].
Hygiene and contact surfaces	Cross-contamination, material degradation, or ineffective cleaning.	Planned: validated cleaning agents, contact-surface instructions, turnover checklist, and material compatibility. Cleaning duration and validation evidence are unavailable.	Cleaning validation, surface inspection, durability testing, infection-control review [42,43,58].
Emergency and clinical deterioration	Delayed response to pain, autonomic symptoms, fatigue, distress, or equipment fault.	Implemented: touchscreen/remote stop, emergency button and magnetic pull cord, support retention and power-loss release. Planned: clinical screening, symptom thresholds, rescue drills, and competency verification.	Scenario-based simulation, staff competency assessment, emergency drills, technical-file audit [18,42,43,44,45,47,50].

Abbreviations: AI, artificial intelligence. Risk management should be integrated with the formal medical-device development process and updated as verification evidence becomes available [42,43].

**Table 4:** Future validation matrix for engineering, usability, and clinical development. Progression should depend on predefined acceptance criteria and documented residual risk.

Development Gate	Validation Domain	Suggested Method	Primary Output or Progression Criterion
1. Specification freeze	Intended use, system boundaries, and traceable requirements	Define users, setting, modes, contraindications, essential performance, interfaces, software functions, and risk controls.	Controlled requirement set with bidirectional traceability and frozen prototype configuration.
2. Bench verification	Mechanical, electrical, lifting, and treadmill–exoskeleton performance	Test static/dynamic loads, joint travel, alignment, speed/acceleration, emergency stop, faults, and electrical/electromagnetic safety.	Essential mechanical and electrical requirements met, including safe-stop behavior.
3. Module verification	Infrared, multisensory, software, cybersecurity, and AI-supported functions	Characterize IR-A output, right–left alternation, phase timing, and thermal safeguards; verify software states, logging, cybersecurity, and bounded AI outputs.	Verified exposure envelope, synchronization tolerance, secure software release, logs, override, and safe degradation.
4. Human factors	Clinician usability, workflow, alarms, training needs, and foreseeable misuse	Conduct independent clinician/engineer appraisal and formative/summative simulated-use tests covering fitting, setup, alarms, AI review, burden, interruptions, and recovery.	Document clinical relevance, task success, critical errors, workload, and training needs; resolve unacceptable use-related risks. No expert/user evaluation has been conducted.
5. Authorized human testing	Basic tolerability, first-in-human safety, and technical feasibility	Proceed only when justified and authorized; prespecify thermal-sensation eligibility, consent, adverse-event monitoring, stopping rules, and a primary feasibility endpoint.	No unacceptable device-related risk; feasible fitting, session completion, tolerance, and data capture.
6. Controlled clinical research	Comparative effectiveness, mechanisms, implementation, and economic value	Use dose- and contact-matched controlled studies; compare the same mechanical platform with and without optional modules. Prespecify outcomes, component contrasts, and justified sample size.	Effect estimates, mechanism signals, durability, implementation feasibility, and evidence for intended claims.

Abbreviations: AI, artificial intelligence; IR-A, infrared-A. Human testing must not begin until the required technical, ethical, and regulatory authorizations are in place.

## Data Availability

No new data were created or analyzed in this concept-level study. Data sharing is therefore not applicable. The design-level specifications supporting the article are reported in the manuscript and Supplementary Materials.

## Supplementary Materials

The following supporting information can be downloaded at: https://www.mdpi.com/article/10.3390/healthcare14183101/s1, Table S1: Extended engineering requirement matrix; Table S2: Extended 11-stage validation checklist; Table S3: Risk-management checklist; Table S4: Infrared characterization protocol; Table S5: Software and AI verification plan; Table S6: Clinician usability protocol.

## Abbreviations

The following abbreviations are used in this manuscript:

AI	Artificial intelligence
BWS	Body-weight support
HMI	Human–machine interface
IFU	Instructions for use
IR	Infrared
IR-A	Infrared-A
ROM	Range of motion
TRP	Transient receptor potential
VR	Virtual reality
