# Supplementary material for: A Multimodal Therapist-Supervised Robotic Gait-Training Platform with Phase-Synchronized Infrared Thermal Biofeedback: A Concept-Level Technical and Translational Healthcare Framework
Source: Healthcare (Basel). 2026 Sep 20;14(18):3101. doi: 10.3390/healthcare14183101 (PMC13606179; doi:10.3390/healthcare14183101)
Supplement: Supplementary file 1 [file healthcare-14-03101-s001.zip › healthcare-4517213-supplementary.pdf]

Supplementary Materials

Table S1. Extended engineering requirement matrix

Implementation status, nominal specifications and unverified design requirements are distinguished from unavailable parameters. “To be determined” denotes parameters without available values; “not measured” denotes absent performance data. No formal or informal bench dataset is available; factory subsystem conformity checks do not verify integrated performance.

Requirement domain	Current implementation or nominal input	Verification needed	Expected evidence	Limitations and unavailable information
Intended use and clinical context	Concept-level use as a therapist-supervised fixed robotic gait-training platform for neurological gait rehabilitation.	Define intended user groups, first target population, setting, operator profile, contraindications and operating modes.	Approved intended-use statement and traceable requirement set.	Target clinical indication and first human-testing population remain to be finalized.
System boundary	All listed modules installed: exoskeleton, treadmill, BWS, optional IR-A, audio, display, RGB camera, touchscreen and AI. Video-live and avatar modes implemented; silhouette planned.	Create system boundary diagram and interface control document; define accessory-module dependencies and stop hierarchy.	Complete system architecture file with module priority and permitted state transitions.	Clock, communication protocol, control cycle, drift, packet-loss and recovery documentation unavailable. Communication-loss shutdown is an unverified requirement.
Mechanical exoskeleton	Four sagittal active axes: bilateral hip/knee flexion-extension; independent electric linear actuators with in-series tension/compression load cells and position sensors. Passive, uninstrumented ankle. Hip ROM/offset, knee flexion ROM (offset ≥0°), speed, cadence, side-specific guidance and BWS are selectable.	Characterize numerical ROM, force/torque and speed limits, sensor resolution and alignment. Verify the design requirement of ±2° ROM accuracy at 100% guidance; it is not measured performance.	Bench-verified mechanical envelope and essential-performance report.	Load-cell component declarations: combined error ≤±0.05% full scale; sensitivity 2 mV/V; thermal drift 0.005%/°C; creep 0.03% at 20 min; IP65; OIML R60. None verified on Li-Walk. Factory-calibrated load cells; unloaded offset zeroing at session start; patient-specific calibration to be determined.
Treadmill synchronization	Motorized treadmill operated with exoskeleton-guided stepping.	Measure belt speed accuracy, acceleration/deceleration, stopping distance, phase coordination and fault behavior.	Synchronization tolerance and safe-stop performance documented under normal and fault states.	Sampling rates: to be determined (undocumented/uncharacterized). Timing metrics: not measured. Gait phase follows the imposed exoskeleton pattern; no validated independent event detector is reported.
Dynamic body-weight support	Dynamic lifting system with load cell and adjustable unloading (85 kg dynamic exercise capacity; 135 kg static lifting capacity).	Verify load-cell accuracy, unloading response, static/dynamic load limits, harness pressure and transition behavior.	Validated unloading envelope and safe-lifting documentation.	85 kg is the dynamic unloading-system exercise rating; 135 kg is the static transfer/positioning rating. Eligible user-mass/unloading combinations and response accuracy require verification.
Anthropometric fit	User height <200 cm and body mass <135 kg are current specifications.	Define segment-length ranges, joint-alignment tolerances, cuff-pressure limits and exclusion criteria.	Fit envelope based on anthropometry and joint alignment, not height alone.	Minimum height and limb-length limits are not reported.
IR-A source characterization	Component declarations: three 200 W halogen lamps per panel (600 W summed ratings), three reflectors, grid/timer, nominal peak near 1.0 µm, 2400-2500 K and full emitter power <1 s. This is not measured output dose or end-to-end latency.	Perform spectroradiometry, irradiance mapping, thermal mapping, exposure-distance testing and duty-cycle characterization.	Source characterization report with exposure envelope and photobiological-risk classification.	Distance, illuminated area, intensity-to-output mapping, maximum cue duration, minimum inter-cue interval and exposure limits: to be determined. Spectrum, irradiance, latency, jitter, shutdown and residual heat: not measured.
IR-A phase synchronization	Implemented: ipsilateral hip-cell force sign/amplitude relative to passive reference at 100% guidance authorizes that side during hip extension at <100% guidance. No EMG, motor-current or kinematic-deviation IR-A trigger input.	Define hip-extension event, interaction-force trigger threshold, trigger latency, jitter, right-left mapping and lockout rules.	Phase timing and false-trigger rate within predefined tolerance.	Firmware feature definition, sampling rate, threshold, window and false-trigger suppression: to be determined (undocumented/uncharacterized). Wrong-sided output has no automatic detector. Excessive deviation causes stop.

Requirement domain	Current implementation or nominal input	Verification needed	Expected evidence	Limitations and unavailable information
Thermal safety	Emitter grid/timer and DIN EN 62471 testing/CE0044 Class IIa status are manufacturer-reported; branch disable and alternative visual/acoustic feedback are available.	Confirm risk group, exposure limits, skin/eye safeguards, temperature cutoffs, residual-risk controls and maintenance checks.	Thermal safety file and worst-case exposure analysis.	No independent temperature sensors installed. Screening, sensory contraindications, monitoring and cutoffs planned; thresholds/maximum exposure: to be determined. Automatic deactivation on stop unverified. Component status does not certify the platform.
Semi-immersive visual interface	49-inch curved monitor presenting synchronized scenarios.	Define resolution, refresh rate, field of view, viewing distance, visual-flow gain, scene-motion limits and latency.	Display characterization and visual-tolerance limits.	Cybersickness profile and latency limits require testing.
Avatar/body representation	Live-video and avatar modes implemented; silhouette planned. RGB video locally processed without recording or network transmission; camera disable available.	Define representation type, latency, perspective, scaling, privacy controls and validation constructs.	Embodiment and privacy validation plan.	Camera model/resolution/frame rate and mapping: to be determined (not documented). Video latency: not measured. Session-data anonymization, re-identification risk, encryption, access and deletion policy unassessed. Video retention not applicable to non-recording mode.
Acoustic feedback	Right-left directional speakers reproduce the corresponding footstep.	Measure sound pressure, timing, laterality, alarm masking, mute behavior and therapist communication preservation.	Auditory timing and sound-pressure verification report.	Alarm priority and sound-pressure limits require testing.
Therapist interface	24-inch touchscreen and remote allow parameter control, pause/stop and AI-independent operation. Key switch, emergency button/magnetic pull cord, manual reset and power-loss release are reported implemented.	Evaluate readability, access control, alarm hierarchy, input errors, override and cleaning compatibility.	Human-machine interface usability evidence for critical tasks.	Usability and foreseeable misuse remain to be assessed.
AI-supported assistant	Rule-constrained case-based software integrated. Inputs: patient profile; speed (km/h), unloading (kg/% body weight), guidance (%), pattern, duration (min), interaction force (N), ROM (degrees), cadence (steps/min). Outputs: suggestions and motivational voice feedback. No parameter write access.	Specify patient-profile fields, similarity metric, feature definitions, preprocessing, update window/frequency and missing-data policy. Test rule limits and clinician decisions independently from IR-A trigger verification.	AI verification file with safe degradation and human confirmation rules.	No trained statistical model or populated clinical cohort database. Recovery-time optimization unvalidated; structured audit trail absent. Similarity, feature, update and missing-data rules: to be determined.
Data logging and auditability	Recommendation interface implemented; complete structured audit trail planned, not implemented.	Log measured state, software version, suggestion, clinician decision, final command and faults.	Complete audit-trail format and data-retention plan.	Data governance and update policy remain to be specified.
Cybersecurity and software lifecycle	Digital layer includes software control and AI-supported functions.	Conduct software lifecycle verification, permissions testing, secure update, rollback, backup and cybersecurity testing.	Verified software release with secure update and safe failure states.	Session-data encryption, retention/deletion and update controls are not formalized or verified; local video processing alone does not establish privacy compliance.
Maintenance and service	Manufacturer-declared 24/7 assistance and intervention within 48 h; preventive maintenance by trained technicians and structured manufacturer training. Approximate footprint 3.3 × 1.6 × 2.5 m; mass ~1000 kg. Reference session 20 min preparation +25 min training =45 min, not timed on a cohort.	Define preventive maintenance, calibration schedule, service logs, spare parts, cleaning and downtime reporting.	Maintenance and serviceability plan linked to risk management.	Training hours and acquisition cost unavailable; cleaning/calibration duration, sessions to competence and uptime not measured. €70-150 per session and up to 10 sessions/day are commercial declarations, not measured cost or throughput. Nominal electrical power: 2 kW standard/up to 3.5 kW with IR (not independently measured).

Abbreviations: *AI, artificial intelligence; BWS, body-weight support; IR-A, infrared-A; ROM, range of motion.*

Table S2. Extended 11-stage validation checklist

This checklist expands the six main manuscript development gates into an 11-stage pathway for technical verification, human-factors testing and clinical development.

Stage	Primary objective	Minimum evidence	Go/no-go criterion
1. Specification freeze	Confirm intended use, users, operating modes, contraindications, system boundaries, essential performance and risk controls.	Controlled design-input file; interface control document; requirement traceability matrix.	Frozen prototype configuration and approved requirement set.
2. Mechanical and electrical bench verification	Verify structure, exoskeleton travel, lifting, treadmill behavior, electrical safety and emergency functions.	Static/dynamic loading, fatigue tests, joint travel, speed/acceleration, emergency stop, fault testing and electrical/electromagnetic testing.	Essential mechanical and electrical requirements met within predefined margins.
3. Treadmill-exoskeleton integration	Verify timing, phase coordination, speed transitions and safe stopping.	Timestamped synchronization tests, jitter analysis, acceleration profiles, stop/recovery scenarios and fault injection.	Synchronization tolerance and safe-stop behavior demonstrated under normal and single-fault states.
4. IR-A module characterization	Characterize spectral output, exposure geometry, right-left alternation, phase timing and thermal safety.	Spectroradiometry, irradiance mapping, skin-equivalent thermal mapping, temperature monitoring, worst-case exposure and IEC/EN 62471 documentation.	Validated exposure envelope, cutoff thresholds, contraindications, operator controls and safe behavior during pause, stop and fault states.
5. Multisensory synchronization	Verify timing among thermal, acoustic, visual and avatar-mediated feedback.	Time-stamped event logging, audio latency tests, visual latency tests, right-left cue mapping and state-machine lockout tests.	Multisensory outputs remain temporally congruent and deactivate correctly during pause, stop or fault.
6. Software, interface and cybersecurity verification	Verify software states, human-machine interface, data integrity, permissions, logs, updates and recovery.	Requirements-based testing, integration tests, usability-oriented error analysis, cybersecurity assessment, backup and rollback testing.	Verified software release with traceability, secure update and controlled failure states.
7. AI-supported function validation	Verify rule constraints and case-retrieval behavior independently from interaction-force trigger logic. Define database quality, suitable-profile criteria, robustness and clinician oversight.	No clinical training dataset exists. Future testing should document reference cases, similarity/feature definitions, missing-data rules, unsuitable profiles, subgroup coverage, retrieval errors and operator responses; trained-model methods apply only if such a model is later developed.	Verified no-write-access boundary, bounded suggestions, clinical relevance and safe clinician override; separate verified force-trigger logic. Complete audit trail must be implemented and tested.
8. Clinician human-factors evaluation	Evaluate setup, fitting, alarms, workload, training needs and foreseeable misuse.	Independent clinical/engineering appraisal plus formative and summative usability tasks; record conflicts, expertise, task success, errors, support needs, workload and perceived relevance.	Critical tasks completed safely; residual use errors documented and acceptable.
9. Authorized healthy-volunteer evaluation	Assess basic tolerability only after all relevant technical safeguards and ethical/regulatory requirements are met; define thermal-sensation eligibility before any IR exposure.	Conservative exposure protocol, technical reliability, thermal comfort, visual tolerance and stop-procedure testing.	No unacceptable device-related risk and predefined feasibility criteria met.
10. Target-population safety and feasibility	Assess target-population fitting, tolerability, workflow and technical reliability.	Prospective supervised study with device-related adverse-event monitoring, predefined stopping rules and technical data capture.	Acceptable safety profile, session completion, reliable operation and refined trial parameters.
11. Comparative clinical development and implementation	Estimate comparative effectiveness, mechanisms, durability, implementation and cost.	Dose/contact-matched controlled or randomized comparisons with and without optional modules; primary endpoint and sample size justified prospectively; blinded assessment where feasible; separate functional and mechanistic outcomes.	Effect estimates, mechanism signals and evidence sufficient for intended clinical claims and sustainable deployment.

Abbreviations: *AI, artificial intelligence; IEC, International Electrotechnical Commission; IR-A, infrared-A.*

Table S3. Risk-management checklist

This checklist supports risk identification and pre-human verification. Each operational-control entry identifies reported implementation and planned or unverified controls; none constitutes experimentally verified system-level mitigation.

Risk domain	Foreseeable hazard	Pre-human verification action	Controls and implementation status	Required evidence
Anthropometric fit and alignment	Joint-axis mismatch, cuff pressure, pain, skin injury or forced range of motion.	Define segment-specific fit limits, alignment guides, interface-pressure limits and exclusion criteria.	Implemented: adjustable hip/knee settings and stop for excessive deviation. Planned/undefined: validated segment-fit limits, alignment tolerances, pressure limits and fitting checklist.	Fit studies across intended anthropometry and simulated misuse.
Body-weight support and lifting	Overload, inaccurate unloading, unintended descent or harness injury.	Static/dynamic load testing, load-cell calibration, fatigue testing and transition testing.	Implemented: load-cell-based support and operator-controlled modes; support retention on emergency stop. Unverified: load accuracy, transitions and fault response. Design status of additional restraints/alarms is not documented.	Load accuracy and safe-transition report.
Treadmill-exoskeleton synchronization	Belt and joint-phase mismatch, abrupt acceleration, instability or stumbling forces.	Latency and jitter measurement, acceleration limits, stopping-distance testing and fault injection.	Implemented: coordinated treadmill/orthosis operation. Unverified requirement: sensory-channel shutdown and controlled mechanical stop on communication loss. Shared-clock, drift and recovery details remain undocumented.	Synchronization tolerance report.
Contingent IR-A biofeedback	Delayed, false-positive, false-negative or wrong-sided cue reinforcing the wrong event.	Define hip-extension event, interaction-force trigger threshold, trigger latency, right-left mapping and lockout.	Implemented: ipsilateral hip-cell authorization and manual exclusion. Undocumented/uncharacterized: force feature, threshold, time window and false-trigger suppression. Unverified: pause/stop lockout; wrong-sided output is not automatically detected.	Trigger testing, false-trigger rate and state-machine verification.
IR-A thermal exposure	Stuck-on cue, residual heating after stop, hotspots, wrong-sided output, repeated exposure or unrecognized injury with impaired thermal sensation.	Spectral/irradiance characterization, skin-equivalent thermal mapping, worst-case exposure and cutoff testing.	Implemented: branch disable and emitter grid/timer. Absent/planned: independent temperature sensing, screening and exposure cutoffs. Undefined: dose/distance limits, thresholds and maximum cue duration. Unverified: automatic IR-A stop-state deactivation.	Thermal safety file and residual-risk evaluation.
Visual and avatar interface	Cybersickness, disorientation, visual strain, fear, embodiment mismatch or privacy concerns.	Display characterization, visual-flow limits, latency testing, avatar-mapping validation and privacy review.	Implemented: live-video/avatar modes and camera disable. Planned: validated perspective/latency limits, graded exposure and symptom assessment; video is locally processed without recording or network transmission.	Validated tolerance and embodiment-measure protocol.
Directional audio	Excessive sound level, distraction, alarm masking or misleading timing.	Sound-pressure measurement, timing/laterality testing and alarm-priority testing.	Implemented: directional step-related sound and therapist control. Planned/unverified: sound-pressure limits, alarm priority, timing limits and communication-preserving alternatives.	Auditory safety and usability report.
Software state control	Incorrect state transition, unsafe command, data loss or unresolved fault.	Unit, integration and system tests; state-machine verification; recovery and rollback tests.	Implemented: software interface and manual operation without AI. Planned: structured audit trail. Undefined/unverified: session-data encryption, retention/deletion, secure update, recovery and access-control validation.	Traceability file and system-test report.

Risk domain	Foreseeable hazard	Pre-human verification action	Controls and implementation status	Required evidence
AI-supported recommendations	Inappropriate suggestion from dissimilar or incomplete profiles, biased case selection, unavailable recovery histories, or automation bias despite nominally bounded outputs.	Define and validate rules, features, similarity, database quality, unsuitable/missing profiles and subgroup coverage; test human confirmation, automation bias and planned logging.	Implemented: rule constraints, therapist decision and no AI parameter write access. Planned: structured audit trail and validation of case retrieval. Confidence/rationale displays and out-of-distribution detection are candidate controls, not established capabilities.	AI verification and monitoring file.
Cybersecurity and updates	Unauthorized access, corrupted update, loss of integrity or unsafe configuration.	Cybersecurity assessment, access-control testing, checksum verification and update rollback.	Implemented: software interface and manual operation without AI. Planned: structured audit trail. Undefined/unverified: session-data encryption, retention/deletion, secure update, recovery and access-control validation.	Cybersecurity report.
Hygiene and contact surfaces	Cross-contamination, material degradation or ineffective cleaning.	Cleaning validation, surface inspection and material-compatibility testing.	Planned: validated cleaning agents, contact-surface instructions, turnover checklist and material compatibility. Cleaning duration and validation evidence are unavailable.	Cleaning and infection-control review.
Emergency and clinical deterioration	Delayed response to pain, autonomic symptoms, fatigue, distress or device fault.	Scenario-based simulation, emergency drills and staff competency assessment.	Implemented: touchscreen/remote stop, emergency button and magnetic pull cord, support retention and power-loss release. Planned: clinical screening, symptom thresholds, rescue drills and competency verification.	Simulation log and competency record.

Abbreviations: *AI, artificial intelligence; IR-A, infrared-A; ROM, range of motion.*

Table S4. Infrared characterization protocol

This prospective protocol defines the minimum characterization of the optional short-wave IR-A subsystem. Independent thermal sensors are absent from the current prototype; screening, exposure limits and cutoffs remain to be defined and validated before authorized human exposure.

Protocol section	Specification
Purpose and scope	Prospective bench/simulated-use protocol; not performed. Installed emitter grid/timer and manual disable do not establish system thermal safety. Independent temperature monitoring and cutoffs must be developed and tested before any human IR-A exposure.
Source identification	Record manufacturer, model, serial number, lamp type, number of lamps, nominal power, supply voltage, timer settings, reflector configuration, protective grid, maintenance state and operating mode.
Environmental and setup conditions	Define ambient conditions, operational distances, angles, target geometry and skin-equivalent material; map each selectable intensity to output. These settings and permissible exposure intervals are not currently characterized.
Spectral characterization	Measure spectral distribution using calibrated spectroradiometry or an equivalent validated method. Report peak emission, wavelength band, measurement uncertainty and any optical filters or reflectors that modify output.
Irradiance and exposure geometry	Map irradiance across the target area at clinically relevant distances. Report uniformity, edge fall-off, hot spots, right-left differences and repeatability across repeated activations.
Thermal mapping	Use skin-equivalent material or validated thermal sensors to record temperature-time curves during intended and worst-case exposure. Include repeated step-like activations, continuous-fault scenarios and cool-down behavior.
Trigger and phase synchronization	Measure force-event detection-to-command and end-to-end irradiance/temperature response separately; include jitter, false/missed triggers, wrong-sided commands, shutdown and thermal decay. The <1 s component power-rise declaration does not supply these measures.
Safety controls	Develop and test independent thermal monitoring, maximum cue duration, inter-cue lockout, exposure thresholds and cutoff on stop/fault. Simulate stuck-on output, sensor fault, hotspot, communication loss and wrong-side wiring. Current architecture has no independent temperature sensor or automatic wrong-side detector.
Acceptance outputs	Define numerical acceptance criteria from applicable standards, calibrated metrology and worst-case testing before progression; these criteria remain to be specified. Produce exposure/synchronization reports, uncertainty, residual-risk analysis and prospective exclusions for impaired thermal sensation.

Abbreviations: *IR-A, infrared-A.*

Table S5. Software and AI verification plan

This prospective plan addresses the integrated rule-constrained, case-based software, which has no trained statistical model or populated clinical cohort database. Structured audit logging is planned, not implemented.

Plan section	Specification
Purpose and scope	To define the verification plan for control software, data logging and the AI-supported therapist-facing decision-support layer. The plan applies before any patient-facing claims are made.
Configuration and version control	Record hardware, firmware, rule-engine and case-retrieval software versions, configuration, database schema and history. There is currently no trained statistical model; record a model version only if one is later developed.
Software state-machine verification	Test setup, calibration, active stepping, pause, controlled stop, fault and emergency states. Confirm that accessory modules cannot remain active in incompatible states.
Data acquisition and logging	Verify sensor provenance, sampling rate, timestamps, missing-data behavior, input validation, clock synchronization, event logging and export integrity.
AI-supported decision support	Formalize profile/feature fields and units, similarity metric, eligible reference cases, temporal/update rules and missing-data handling. Test rule constraints, inappropriate retrieval, bias and clinician usefulness. Recovery-time comparisons require a clinical database and defined outcomes before evaluation.
Human confirmation and override	Test that suggestions cannot write treatment parameters; assess accept/modify/ignore workflows and automation bias. Implement the planned structured audit trail linking inputs, proposal, clinician decision and final command before claiming traceable use.
Cybersecurity and update control	Verify local non-recording video behavior separately from session-data handling. Establish de-identification, access, encryption, retention/deletion and secure updates; test leakage, unauthorized access, rollback and recovery.
Failure and safe degradation	Simulate sensor dropout, delayed data, inconsistent state, failed AI module, network failure, corrupted log and power interruption. Verify safe degradation and basic manual mode where applicable.
Acceptance outputs	Produce a verified software release file, AI validation report, audit-trail specification, cybersecurity report, safe-degradation report and unresolved-risk register.

Abbreviations: *AI, artificial intelligence.*

Table S6. Clinician usability protocol

This prospective protocol combines independent expert appraisal and simulated-use evaluation before patient exposure. No clinician evaluation has been performed. Future recruitment requires the applicable ethics determination, informed consent and controlled handling of participant data.

Protocol section	Specification
Purpose and scope	To evaluate clinician-facing usability and human factors in simulated-use conditions before patient exposure. The protocol is not a clinical effectiveness study.
Participants	Recruit representative physiotherapists/rehabilitation clinicians and independent rehabilitation engineers for expert appraisal; report roles, experience, prior device exposure and commercial relationships. Record training and obtain appropriate ethics determination and consent.
Training materials	Provide standardized instructions, safety checklist, fitting guide, alarm guide, parameter guide, AI-suggestion interpretation guide and cleaning/turnover instructions.
Critical tasks	Assess device startup, user selection scenario, fitting, alignment, BWS calibration, parameter selection, scenario selection, start/pause/stop, response to alarms, emergency stop, override of AI suggestion and session closure.
Simulated-use scenarios	Use normal and interrupted setup, poor fit, mode confusion, simulated synchronization/thermal faults and inappropriate AI suggestions. Thermal scenarios must use simulation/phantoms until safeguards permit exposure. Include camera refusal and sensory-channel exclusion.
Outcome measures	Record task success, critical/non-critical errors, time, assistance requests, workload, clarity, learnability and clinical relevance. Device sensing supplies force/position-related signals only after validation; calibrated external instrumentation is needed for timing/thermal accuracy, EMG for muscle activation, questionnaires for agency/workload, and authorized clinical assessment for functional outcomes.
Acceptance criteria	Prespecify critical-error definitions, acceptable task performance and unresolved-hazard rules before testing. Analyze technical reliability, safety, sensorimotor behavior, user experience, clinical function and implementation separately. Expert ratings are feasibility/relevance evidence, not clinical efficacy.
Data handling	Use de-identified clinician data. Store task logs, observation sheets, questionnaire results and comments in a controlled file linked to software and device configuration.
Reporting outputs	Report independent appraisal, disagreements, observed use errors, workload, training needs, root causes and corrective actions. Subsequent clinical studies should prespecify primary outcome, matched comparator, dose/contact, sample-size rationale and blinded assessment where feasible.

Abbreviations: *AI, artificial intelligence; BWS, body-weight support.*
